# Bone and Joint Infections due to* Haemophilus parainfluenzae*: Case Report and Review of the Literature

**DOI:** 10.1155/2016/4503025

**Published:** 2016-07-19

**Authors:** Conar R. O'Neil, Evan Wilson, Bayan Missaghi

**Affiliations:** ^1^Department of Internal Medicine, Cumming School of Medicine, Health Sciences Centre, University of Calgary, Foothills Campus, 3330 Hospital Drive NW, Calgary, AB, Canada T2N 4N1; ^2^Department of Microbiology, Immunology and Infectious Disease, Cumming School of Medicine, Health Sciences Centre, University of Calgary, Foothills Campus, 3330 Hospital Drive NW, Calgary, AB, Canada T2N 4N1

## Abstract

*Haemophilus parainfluenzae* is a normal inhabitant of the human respiratory tract. However it is an increasingly recognized pathogen in invasive infections, particularly in the immunocompromised host and where there is disruption of the normal skin or mucosal barriers. We present a case of a 56-year-old female with a history of asplenia who developed* H. parainfluenzae* septic arthritis of the hip following an intra-articular steroid injection. We also summarize previously reported cases of bone and joint infections caused by* H. parainfluenzae*.

## 1. Case Presentation

A 56-year-old female presented to the emergency room with a three-day history of right hip pain. She reported decreased range of motion and difficulty ambulating. She denied constitutional symptoms or fever. She had undergone intra-articular steroid injection of her right hip three days prior. Review of systems was otherwise unremarkable. Her past medical history was relevant for hereditary spherocytosis with splenectomy at age 14 and chronic right hip osteoarthritis. Her only medication was celecoxib as needed. She denied any drug allergy. She was unaware of her immunization history. She worked as a flight attendant, denied smoking or recreational drug use, and had not recently travelled outside of Canada. On initial examination she was afebrile and her vital signs were within normal limits. On examination of her right hip she had limitation of internal rotation with reproducible pain, but her physical exam otherwise was unremarkable. Her initial investigations showed elevation in C-reactive protein (CRP) and erythrocyte sedimentation rate (ESR) at 22.9 mg/L and 25 mm/hr, respectively. Her white blood cell count was not elevated. An X-ray of her right hip showed severe joint space narrowing with osteophytosis consistent with severe osteoarthritis. Arthrocentesis was performed and synovial fluid samples were directly inoculated onto solid culture media, including blood agar and chocolate agar. Two of three samples also underwent cytospin centrifugation and direct Gram stain. Gram-negative bacilli were observed in one sample though heavy neutrophils were observed in both. Synovial fluid analysis for cell count and chemistry was not performed due to insufficient sample. She was admitted to hospital for 24 hours of observation and was subsequently discharged with instructions to return to hospital if her symptoms worsened or if cultures subsequently grew a pathogenic organism.

**Figure 1:**
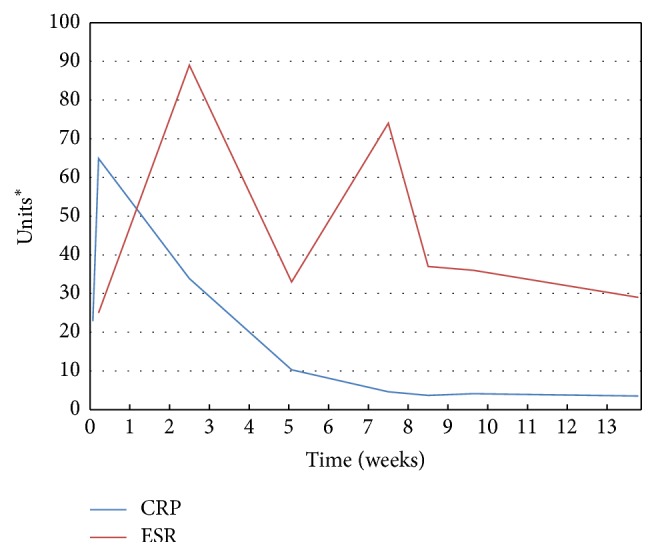
Inflammatory markers over time during a prolonged course of intravenous ceftriaxone for* Haemophilus parainfluenzae* septic arthritis. CRP: C-reactive protein; ESR: erythrocyte sedimentation rate. ^*∗*^CRP = mg/L; ESR = mm/hr.

Three days later she was seen in a follow-up clinic. In the interim she had developed a fever (38.3°C), had chills, and had worsening right hip pain. Repeat physical examination showed deterioration in range of motion at her right hip with significant pain. Cardiovascular examination was unremarkable with no appreciable murmur. Synovial fluid cultures from the initial arthrocentesis were now growing* Haemophilus parainfluenzae* with colonies observed on solid media within 24 hours of inoculation. She received one dose of ceftriaxone and subsequently underwent right hip arthrotomy with synovectomy and irrigation. The Infectious Disease service was consulted the following day. The organism was susceptible to ceftriaxone and cefuroxime and resistant to ampicillin and ciprofloxacin (Table 1). Blood cultures and intraoperative tissue cultures did not demonstrate growth, likely due to the administration of antibiotics prior to collection. The patient was treated with intravenous ceftriaxone and was subsequently discharged with outpatient follow-up for home intravenous antibiotics. Ongoing pain, difficulty ambulating, and persistently elevated inflammatory markers necessitated a prolonged, nine-week course of antimicrobial therapy (Figure 1). At the time of treatment discontinuation, she reported pain and her functional ability had not yet returned to baseline. She is currently awaiting evaluation for total hip arthroplasty.

## 2. Discussion

*H. parainfluenzae* is a pleomorphic Gram-negative coccobacillus with fastidious growth requirements, which require enriched media, usually containing blood (e.g., chocolate agar). It can be differentiated from other* Haemophilus* spp. by the requirement for V factor (i.e., NAD, nicotinamide adenine dinucleotide) for growth [1].* H. parainfluenzae* is part of the normal flora of the oral cavity and respiratory tract [1]. It is an increasingly recognized opportunistic pathogen in serious infections such as endocarditis, meningitis, and pneumonia and has also been recognized as a rare cause of nongonococcal urethritis [2]. However, it is an uncommon pathogen in osteomyelitis and septic arthritis.

Inclusive of our case, there have been only 16 cases of bone and joint infections caused by* H. parainfluenzae* reported in the English literature (summarized in Table 2) [3–16]. Of these 16 patients, 10 (63%) had septic arthritis, four (25%) had osteomyelitis, and two (13%) had both septic arthritis and osteomyelitis. The median age was 65 (Interquartile Range [IQR]: 46–76) and 63% were male. Most (14/16, 88%) were immunocompetent and only one reported recent trauma [14]. Four patients (25%) had prosthetic joint infections. Most patients (10/16, 63%) reported a procedure in the previous three months (four dental, two nasopharyngeal, two gastrointestinal, one total knee arthroplasty, and one intra-articular steroid injection). The organism was identified on culture of aspirated synovial fluid (6/16, 38%), blood (4/16, 25%), and surgical or biopsy specimens (7/16, 44%). 8/16 (50%) of patients required surgical intervention and the rest (8/16, 50%) were managed with antibiotics alone. One patient with a prosthetic joint infection was treated with chronic, suppressive antibiotic therapy after having refused surgical intervention [10]. The median length of antibiotic therapy was 42 days (IQR: 14–70). Of patients with data reported, most (11/14, 79%) were treated with a beta-lactam antibiotic, the most common being ampicillin (7/14, 50%).

To our knowledge, we report the first case of septic arthritis due to a beta-lactamase producing strain of* H. parainfluenzae* (Table 1). Community surveillance studies of* Haemophilus* spp. isolated from respiratory samples have reported rates of beta-lactamase production as high as 70% [17, 18]. Beta-lactamase plasmids can transmit from less pathogenic strains of* Haemophilus*, such as* H. parainfluenzae*, to invasive pathogens such as* Haemophilus influenza* type b, suggesting* H. parainfluenzae* may be a reservoir for antimicrobial resistance in the nasopharyngeal tract [18]. More recently, increasing rates of antimicrobial resistance have also been reported in genitourinary isolates of* H. parainfluenzae* [2, 19].

Moreover, it is well known that immunocompromised hosts are particularly susceptible to opportunistic pathogens, and it is within this context that we report the first case of septic arthritis due to* H. parainfluenzae* in a patient with asplenia. Patients with asplenia are at significant increased risk of sepsis due to encapsulated bacteria, such as* Haemophilus* spp.,* Neisseria* spp., and* Streptococcus pneumoniae* [20]. Immunizations for pneumococcal disease,* Haemophilus influenzae* type b infection, and meningococcal disease are recommended for asplenic patients. However, they remain susceptible to less common potentially pathogenic bacteria, such as* H. parainfluenzae*. Prompt recognition and source control (e.g., surgical debridement) are particularly important in preventing disseminated infection in patients with asplenia. In our case, given her risk of disseminated infection, surgical intervention and antibiotics could have been initiated sooner, when Gram stain of the initial arthrocentesis was reported.

To the best of our knowledge, our case is the only example of* H. parainfluenzae *septic arthritis temporally associated with intra-articular steroid injection that is described in the literature. Septic arthritis is a rare but recognized complication following intra-articular steroid injection. Infection may result from lack of adherence to proper aseptic technique during the procedure and subsequent inoculation of the joint space, or through microbiological contamination of the steroid preparation used [21, 22]. Alternatively, transient bacteremia can lead to secondary seeding of a susceptible joint capsule, for example, in patients with underlying osteoarthritis or previous joint infection. However, localized immunosuppression from intra-articular steroid injections does not appear to be associated with increased rates of deep infection as demonstrated in patients receiving steroid injection prior to joint arthroplasty [23, 24].

*H. parainfluenzae* is an uncommon though increasingly recognized pathogen in bone and joint infections. In this series, patients with prosthetic joints and patients undergoing invasive procedures appear to be particularly susceptible to this pathogen. Furthermore, the rate of beta-lactamase producing strains of* H. parainfluenzae* may be increasing. Thus, accurate microbiologic diagnosis is key to providing tailored antibiotics, as patients often require a prolonged course of therapy.

## Figures and Tables

**Table 1:** *Haemophilus parainfluenzae* susceptibility results.

Antibiotic	Result	Method
Beta-lactamase	Positive	Nitrocefin SR112, Oxoid Microbiology Products
Ciprofloxacin	Resistant	MIC by Etest = 6 ug/mL Sensitive ≤ 1
Ceftriaxone	Susceptible (32 mm)	Kirby Bauer
Cefuroxime	Susceptible (30 mm)	Kirby Bauer
Meropenem	Susceptible (28 mm)	Kirby Bauer

Disk diffusion and Etest as per CLSI M100 S24 document; *Haemophilus* test medium: 35°C ± 2°C in 5% CO_2_ for 16–18 hours.

**Table 2:** Summary of previously reported cases of bone and joint infections caused by *Haemophilus parainfluenzae*.

	Age/sex	Site of infection	Prosthetic joint	Procedure^*∗*^	Comorbidities	Positive cultures	Surgical intervention	Beta-lactamase	Antibiotics	Length of therapy	Outcome
Our case	56 F	Hip	No	Interarticular steroid injection	Asplenic	Synovial fluid	Arthrotomy	Positive	Ceftriaxone	9 weeks	Cure
Hong et al. [3]	53 M	AC joint^1^	No	None	None	Blood	No	Not reported	Cefazolin and gentamicin	4 weeks	Cure
Bailey et al. [4]	75 M	Knee	Yes	TKA^3^	CLL^4^	Tissue	2-stage revision of TKA	Negative	Flucloxacillin and rifampicin	10 weeks	Cure
Carey et al. [5]	60s F	AC joint, clavicle	No	None	None	Tissue	Incision and drainage	Not reported	Levofloxacin	14 days	Cure
Khor et al. [6]	79 M	Spine, epidural, psoas		Gastroscopy	None	Tissue	Laminectomy	Negative	Ampicillin	14 weeks	Cure
	36 M	SI joint^2^, spine		None	None	Blood	No	Negative	Ampicillin	10 weeks	Cure
Jellicoe et al. [7]	78 F	Hip	Yes	Dental	None	Synovial fluid and tissue	2-stage revision of THA^7^	Negative	Ampicillin and flucloxacillin	4 weeks	Cure
Blanche et al. [8]	26 M	Knee	No	Nasopharyngeal biopsy	HIV^5^	Blood and synovial fluid	No	Not reported	Not reported	Not reported	Cure
Beauvais et al. [9]	70 M	Spine		Gastroscopy and colonoscopy	Colon cancer	Tissue	No	Not reported	Not reported	Not reported	Cure
Manian [10]	72 M	Knee	Yes	Dental	None	Wound swab	No	Negative	Ciprofloxacin	Chronic, suppressive	Chronic infection
Auten et al. [11]	74 M	Spine, epidural		Dental	None	Tissue	Laminectomy	Negative	TMP/SMX^8^ and tobramycin	7 weeks	Cure
Pravda and Habermann [12]	78 F	Knee	Yes	Dental	None	Synovial fluid	Arthrotomy	Negative	Ampicillin and amoxicillin	12 weeks	Cure
Olk et al. [13]	49 M	Spine		Nasal septoplasty	None	Tissue	No	Negative	Ceftriaxone	6 weeks	Cure
Warman et al. [14]	95 F	Ankle, meningitis	No	None	Dementia	Synovial fluid and CSF^6^	No	Negative	Ampicillin	14 days	Cure
Oill et al. [15]	18 M	Polyarticular	No	None	None	Blood	No	Not reported	Penicillin and ampicillin	7 days	Lost to follow-up
Renne et al. [16]	8 mo F	Knee	No	None	Otitis media	Synovial fluid	Arthrotomy	Not reported	Ampicillin	10 days	Cure

^*∗*^In the previous three months; ^1^acromioclavicular joint, ^2^sacroiliac joint,^ 3^total knee arthroplasty, ^4^chronic lymphocytic leukemia, ^5^human immunodeficiency virus, ^6^cerebrospinal fluid, ^7^total hip arthroplasty, and ^8^trimethoprim/sulfamethoxazole.
